# Polymer Coated Functional Catalysts for Industrial Applications

**DOI:** 10.3390/polym15092009

**Published:** 2023-04-24

**Authors:** Raj Kumar Arya, Devyani Thapliyal, Anwesha Pandit, Suchita Gora, Chitrita Banerjee, George D. Verros, Pramita Sen

**Affiliations:** 1 Department of Chemical Engineering, Dr. B.R. Ambedkar National Institute of Technology, Jalandhar 144011, India; devyanithapliyal5@gmail.com; 2 Department of Chemical Engineering, Heritage Institute of Technology, Kolkata 700107, India; anwesha.pandit.che23@heritageit.edu.in (A.P.); suchita.gora.che24@heritageit.edu.in (S.G.); chitrita.banerjee.che24@heritageit.edu.in (C.B.); 3 Laboratory of Polymer and Colour Chemistry and Technology, Department of Chemistry, Aristotle University of Thessaloniki, Plagiari, Epanomi, P.O. Box 454, 57500 Thessaloniki, Greece; gdverros@gmail.com

**Keywords:** catalytic activity, polymeric coating, productivity, biopolymer, functional coatings

## Abstract

Surface engineering of conventional catalysts using polymeric coating has been extensively explored for producing hybrid catalytic material with enhanced activity, high mechanical and thermal stability, enhanced productivity, and selectivity of the desired product. The present review discusses in detail the state-of-the-art knowledge on surface modification of catalysts, namely photocatalysts, electrocatalysts, catalysts for photoelectrochemical reactions, and catalysts for other types of reactions, such as hydrodesulfurization, carbon dioxide cycloaddition, and noble metal-catalyzed oxidation/reduction reactions. The various techniques employed for the polymer coating of catalysts are discussed and the role of polymers in enhancing the catalytic activity is critically analyzed. The review further discusses the applications of biodegradable and biocompatible natural polysaccharide-based polymers, namely, chitosan and polydopamine as prospective coating material.

## 1. Introduction

The enhancement of industry-scale chemical processes in terms of high yield, productivity, selectivity, and purity of a product requires the development of novel catalysts. Rationally engineered hybrid material for catalytic applications has shown excellent performance in terms of enhanced catalytic activity, selectivity, lifetime, and recyclability of catalysts [1]. Recent years have witnessed the development of polymeric material as a novel catalyst, catalyst supports, and surface modification material due to the ease of production/catalyst separation [2], enhancement of selectivity, conversion, catalyst recyclability, and improved moisture resistance of the catalyst through the incorporation of hydrophobicity on the catalyst surface [3]. For instance, polydimethylsiloxane-coated metal oxide catalysts exhibit superhydrophobic characteristics, preventing deactivation of the catalyst [4]. Polymer-coated ceria nanocatalysts showed remarkable improvement in catalytic activity through fast oxidation of dyes and also exhibited resistance to denaturation and decomposition [5]. Metallic nanoparticle-based catalysts, namely, silver, gold, platinum, palladium, zinc, and copper nanocatalysts, are widely used in reactions due to their higher catalytic activity offered by large surface–volume ratio. However, nanocatalysts suffer from certain limitations, such as difficulty in catalyst recovery and loss of activity due to aggregation. To overcome these challenges, various modifications have been made in existing nanocatalysts. These include immobilization of transition metal-based nanocatalyst particles in magnetic nanoparticles, followed by coating with polymeric material or direct coating of catalyst particles with a polymeric coating [6], which have been effectively implemented in carrying out organic synthesis reactions with enhanced catalytic activity [7].

A promising technological approach for creating multilayered assemblies and managing the surface characteristics of electrocatalysts is the use of polymeric coatings on catalysts. The microenvironment of polymeric coatings promotes charge transfer to redox active sites, substrate diffusivity, and chemical protection for the solid support material and embedded catalyst [1]. Polymer coating material, in the form of a covalent organic framework, metal organic framework, and surface-bound polymers are applied for encapsulation of catalytic material widely used in electrocatalytic and electrophotosynthetic applications through multilayered molecular functionalization. These coating materials are either covalently tethered to electrodes or deposited as insoluble films. Several studies have reported the use of polymer coatings for modification of electrode/electrolyte interfaces and for tuning of surface electronic properties of electrocatalysts [8]. Platinum nanoparticles with a capping of non-electroactive cationic polyelectrolyte, such as polydiallyldimethyl ammonium chloride (PDDA) assembled into layer-by-layer arrays have been successfully employed in electrocatalysis of oxygen reduction reactions and hydrogen evolution under potential deposition [9]. Furthermore, platinum nanoparticles capped using polyvinylpyrrolidone (PVP) and assembled in LBL arrays showed an enhancement of formic acid and methanol oxidation reactions [10].

The coating of conducting polymers on metal oxide photocatalysts produces a bandgap in the visible region and the coated catalysts have been effectively used for photocatalytic degradation of dyes under visible solar irradiation with enhanced stability [11].

Nature-inspired biopolymer coatings have recently been employed for the coating of supported metal and metal oxide-based catalysts for improvement of catalytic performance [12]. Polydopamine (PDA)/nickel complex-coated multiwalled carbon nanotubes are used as combustion catalysts for decomposition and combustion of energetic material [13]. Biopolymers like chitosan have been effectively employed for coating metal organic framework-based catalyst nanoparticles [14] and metal oxide-based nanoparticles used for electrocatalysis [15].

The present review explores the necessity of applying polymer functional coatings on different types of catalysts. The review provides a detailed discussion on the state-of-the-art knowledge on functional polymer-based surface modification techniques of catalysts employed in different types of reactions. The review begins with a discussion about the different types of polymers used for the functional coating of catalysts. In the subsequent sections, brief descriptions of the methods used for the preparation of polymer-coated catalysts are provided. The applications of polymer-coated catalysts in different types of reactions are enumerated and the effects of polymer coating on the activity, selectivity, stability, durability, and reusability of the catalysts are discussed. Special emphasis has been put on analyzing the applications of polymer-coated catalysts in photocatalytic, electrocatalytic and electrophotosynthetic reactions. The effects of coating on the reactant conversion/pollutant degradation efficiency, product yield and selectivity, recyclability of the catalyst, and thermal and mechanical stability of the polymer/catalyst composites are outlined. The role of mass transfer (diffusion) limitations, if any, on the performance of polymer-coated catalysts is also discussed in detail. Finally, a brief discussion on the application of nature-inspired biopolymers, namely, chitosan and polydopamine, as functional coating material for enhancing catalytic activities is provided.

## 2. Types of Polymers Used as Coating Material for Catalysts

Based on their source, polymers can be classified as synthetic, semi-synthetic, or natural/biopolymers. Of the three classes of polymers, synthetic and natural polymers have been extensively used as coating material for catalysts and other applications.

### 2.1. Synthetic Polymers

Synthetic polymers are produced from their basic building blocks, namely, monomeric units obtained mainly from the petroleum and petrochemical industries. The selection of polymeric material for the coating of catalysts must be governed by several factors, including mechanical and thermal stability, enhanced activity, selectivity for the desired product, increased lifetime, and recyclability. The synthetic polymers used as coating material are subclassified based on the method of synthesis as follows.

#### 2.1.1. Step-Growth Polymers

Synthetic polymers produced by step-growth polymerization include polyesters, polyamides, polyurethanes, polyether, and polyurea. The semicrystalline polymer, polyamides, have low coefficients of friction, high resilience, and excellent wear properties and are preferred as a coating material. Polysiloxanes are thermally stable and UV-resistant polymers with low glass transition temperatures and are also preferred industrially as a protective coating material. Polysiloxane gels encapsulating a platinum catalyst have been used multiple times to reduce nitroarenes to anilines [16]. A hyperbranched thermoset polymer, namely, polyester, prepared by polycondensation method along with TiO_2_ nanowires were employed to prepare polyester/TiO_2_ nanohybrids, whereby good dispersion of nanoparticles in the polymer matrix was achieved. The nanocomposites achieved 70% removal of COD from waste effluent without degradation due to a smaller crystallite size [17]. TiO_2_ nanoparticles were deposited on 3D-printed polyamide open structures and the composite was employed for dye removal in a 360-degree rotating photocatalytic reactor to maximize the irradiance distribution throughout the reactor and achieve a high percentage (90%) of dye removal [18]. Specialty polymers, namely, polyarylene sulfides, are preferred as a protective coating material because of their excellent mechanical and thermal stability. Polynitrogen containing nonconjugated crosslinked polymer, poly(1,4,8,11-cyclotetradecane [2,2-bipyridine]-5,5-dicarboxamine (PNH), has better chemical stability compared with conjugated polymers that form a polymeric metal complex, PNH-Fe(III) with the photo-Fenton material Fe_2_O_3_, owing to its strong coordination affinity with Fe(III), and the composite material has been employed for pollutant removal [19]. In recent years, photografting of hydroxyl ethyl methacrylate (HEMA) onto graphitic carbon nitride (g-CN) generated well-dispersed g-CN precursor colloids, cyanuric acid-melamine (CM)-HEMA, which were mixed with cross-linkers, such as citric acid, to produce a stable coating. The coating was further functionalized via the grafting of the polymers, namely, polystyrene (PS) or polydimethylacrylamide (PDMA), and was effectively used for photocatalytic degradation of dye [20].

#### 2.1.2. Chain Growth Polymers

Polymers synthesized by chain growth polymerization, in which unsaturated monomers add to the active sites (free radicals) of the growing polymer through the initiation, propagation, and termination steps, include polyethylene, polypropylene, polyvinyl chloride, polymethyl methacrylate, polyvinyl acetate, and polyvinyl alcohol. Polyvinyl alcohol has been used for the coating of tablets in the form of thin films and has also been used for pharmaceutical applications. In recent years, three-dimensional microporous perovskite-type strontium or lanthanum oxides in polymethyl methacrylate (PMMA) or polystyrene (PS) microspheres as the template have been employed to remove VOCs through combustion reactions [21]. PMMA-capped aluminum nanoparticle composites have been applied for photolysis reactions with 100% retention in catalytic activity [22].

#### 2.1.3. Coordination Polymers

Recently, coordination polymers, another class of synthetic polymers, including covalent organic frameworks (COF) and metal organic frameworks (MOF), have emerged as powerful coating material to be used in electrocatalysis and photoelectrosynthesis [1]. For instance, in metal organic frameworks, the organic units, namely, carboxylates, are linked to the metal-containing units yielding robust crystalline structures with a surface area in the range of 1000–100,000 m^2^/g and high porosity. MOFs have been widely employed as electrocatalytic and electrophotosynthetic coating material. In the area of electrocatalytic coating, the MOF-based catalysts constructed with zirconium-based nodes that coordinate with iron/carboxyphenyl porphyrin functional groups have been used as coating material for electrodes in oxygen reduction reactions [23], producing less than 6% peroxide, indicating high H_2_O/H_2_O_2_ selectivity. Similarly, MOF thin films prepared with Ni-porphyrin linkers and zirconium nodes produced 13 μmol/L oxygen, which was about 4.5 times higher than non-metallated films [24]. MOF containing catalytically active sites linked with semiconductor surfaces resulted in photoelectrochemical assemblies evolving hydrogen from aqueous solutions [25].

Another application of coordination polymers has been observed in the coating of molecular catalysts, in which cobalt porphyrins enclosed in surface-attached organic polymer coatings or in polymeric membranes exhibit superior catalytic activity and product selectivity in carbon dioxide and proton reduction [26,27,28]. Polymeric matrices, such as PMMA used for enclosing chromophore-catalyst assemblies, ensure the surface stability of the catalyst and provide resistance to high-energy radiation and the oxidative degradation during electrochemical reactions [1]. Furthermore, the coordination of catalysts to surface grafted polymers produces assemblies, such as cobalt-polypyridyl thin-film-coated gallium phosphide photocathodes, which have been successfully employed to produce hydrogen from aqueous solutions using solar energy [29].

#### 2.1.4. Conjugated and Non-Conjugated Polymers

Conjugated polymers (CP) with a photoactive π system are extensively employed for solar energy utilization and photocatalysis, including water splitting, carbon dioxide reduction, organic transformation, and dye degradation [30]. Conjugate polymers, including those from the polyfluorene (PF) family, are organic semiconductors with enormous applications in optoelectronics, including polymer solar cells and organic light emitting diodes [31], owing to their enhanced visible light absorption capacity, high hole/electron mobility, and high thermal and electrical stability. PFs are used for photocatalytic applications, especially in solar hydrogen conversion (SHC). Polymeric heterojunctions (PHJ) photocatalysts consisting of PF family polymers and graphitic carbon nitride (g-C_3_N_4_) serve as effective photocatalysts. On the other hand, non-conjugated cross-linked polymers (NCCP) having better chemical stability compared to conjugated polymers and have been designed to overcome the shortcomings of conjugated polymers. The NCCPs, including poly-cyclotetradecane [2,2-bipyridine]-5,5-dicarboxamine (PNH), avoid π–π stacking interactions, resulting in a large number of active electron-donating sites in PNH that can interact with the semiconductor [19].

#### 2.1.5. Conducting Polymers

Conducting polymer/inorganic hybrid materials have been extensively used in enhancing the photocatalytic activity of the existing photocatalyst [32] and enhancing the hydrogen production activity of semiconductors [33]. Polyaniline (PANI), poly(3-hexylthiophene) (P_3_HT), and polypyrrole are commonly used conducting polymers for this purpose. Conducting polymers have an extended π-conjugated electron system and act as a stable photosensitizer to sensitize TiO_2_ by broad spectrum of UV-visible radiation. The combination of PANI, a p-type conducting polymer, and TiO_2_, an n-type semiconductor, possesses good conductivity and stability. P_3_HT–platinum–graphitic carbon nitride (g-C_3_N_4_) composites have been effectively used for hydrogen production from a Na_2_S-Na_2_SO_3_ solution with a 300% increase in hydrogen production activity due to the addition of 3% P_3_HT [34].

#### 2.1.6. Recent Development in Polymer-Based Coating Materials

Apart from the polymer-based coating material discussed in the previous section, in recent years, there has been considerable progress in polymer science and engineering in fabricating novel polymer–catalyst hybrid material. These include hollow polymer nanocapsules as a coating material for catalysts, porous material for encapsulating single atom catalysts, polymer chelation-based catalysts, and paramagnetic nanocomposites of polymer, metallic nanoparticles, and Fe_3_O_4_.

##### Hollow Polymer Nanocapsules

**Figure 1:**
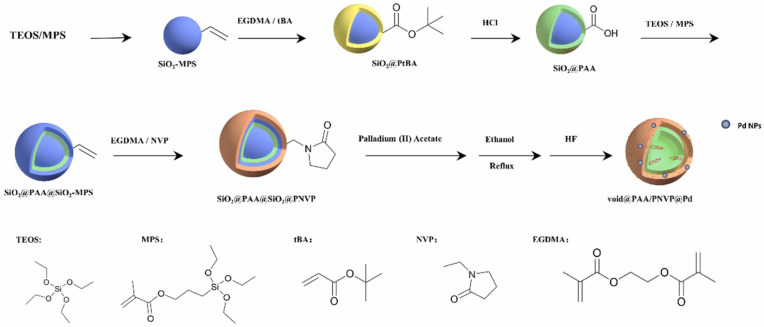
Synthesis of void PAA/PNVP/Pd hollow polymer microspheres (reprinted with permission from [38]).

In recent years, hollow polymer nanocapsules (HPN) comprising a robust shell with hollow pores and unique properties, namely, large surface area, low density, and high loading capacity, have been used for numerous applications, including micro/nanoreactors, catalysts, energy storage material, and material for environmental remediation [35]. Surface-initiated reversible addition fragmentation chain-transfer (RAFT) polymerization has been employed to prepare polymeric nanocapsules using silica nanoparticles as sacrificial templates and a polymeric shell of poly[poly(tert-butylmethacrylate)-co-poly(2,3-dimethylmaleicimidopropylmethacrylate)-b-poly(2-hydroxypropyl methacrylamide)], where the dimethylmaleic amidopropyl methacrylate acts as the photo-crosslinker for stabilizing the nanocapsules [36]. Polymer nanocapsules with pH responsive inner shells and thermoresponsive outer shells can also be stabilized by grafting the nanocapsules onto a silica template [37]. For dual functional frameworks comprising a burn rate catalyst ferrocene and a metallic fuel, an aluminum nanoparticle was selected and embedded within the hollow space of the HPN with a shell consisting of ferrocene [35]. Double-shelled hollow poly(ethylene glycol dimethacrylate-co-acrylic acid) (PAA) and poly(ethylene glycol dimethacrylate-co-N-vinyl pyrrolidone) (PNVP) microspheres with carboxylic acid in the inner shell and palladium metallic nanocolloids in the outer shell (Figure 1) have been used as bifunctional catalysts for tandem reactions, namely, benzaldehyde dimethyl acetal to benzyl alcohol [38].

##### Single-Atom Catalysts (SACs)

Single-atom catalysts (SACs) are recently developed novel catalysts in which a single atom on the catalyst’s surface carries out a catalytic reaction [39,40]. A large number of porous materials, namely, zeolites, metal organic framework, and carbon nitride, are used for encapsulating SACs. A MOF structure (MOF-808) with ethylenediaminetetraacetic acid (EDTA) ligand anchored on the metal node of the Zr_6_ cluster was used to capture a platinum ion, whereby the MOF/EDTA-coated monatomic platinum catalyst exhibited excellent photocatalytic activity [41] with 99% removal of the metal ions. A novel two-dimensional quasi-Fe-Zn-phtalocyanine polymer derived Fe-N-C single atom multifunctional catalyst has been prepared to carry out an oxygen reduction reaction with superior electrocatalytic activity and peroxidase-like activity [40].

##### Polymer Chelation for Preparing Transition Metal-Based Catalysts

Alternatives to platinum catalysts, namely, transition metals, such as iron (Fe), cobalt (Co), nitrogen, and carbon-based electrocatalysts (M-Nx/C) for an oxygen reduction reaction (ORR), have been actively investigated where the nitrogen-coordinated metal sites act as active centers for the ORR [42]. Nitrogen-rich polymers, namely, poly-1,8-diaminonaphthalene (PDAN), has been used as the N, C precursor for the preparation of N-doped carbons with a porous structure, enabling oxygen diffusion and water removal. Based on the concept, a polymer chelation strategy utilizing a PDAN-Fe(III) chelating complex has been used as a precursor for synthesis of Fe-Nx-C, whereby the Fe-Nx active sites are uniformly distributed onto the surface of the carbon spheres [43].

##### Magnetic Particle-Based Catalyst

Magnetic polymer-based nanocatalysts are beneficial for their unique properties, including simple separation and regeneration, high product purity, and selectivity. In one of the studies reviewed, the polymer poly(styrene-co-maleic anhydride) (PSMA) was modified by a cross-linking agent, melamine, followed by sulfonation. Using in-situ co-precipitation, a paramagnetic nanocomposite comprising sulfonated PSMA and Fe_3_O_4_ nanoparticles was prepared. The resulting nanocomposite was used to catalyze the reaction of 4-hydroxycoumarin, aromatic aldehyde, and malononitrile, producing a maximum yield of 92% of the targeted dihydropyranochromenes [44]. The co-precipitation approach has also been used for in situ magnetization of curcumin with Fe_3_O_4_ superparamagnetic nanoparticles. The hybrid unit was functionalized with 3-chloropropyltrimethoxysilane, followed by the attachment of melamine and immobilization of the silver nanoparticles to prepare the Fe_3_O_4_/curcumin/melamine-Ag nanocomposite catalyst for the efficient reduction of nitrobenzene derivatives. The highest yield of 95% in the nitrobenzene reduction has been achieved with 0.02 g of the composite catalyst in the presence of hydrazine hydrate in ethanol during a 10 min reaction time [45]. Composites of Fe_3_O_4_-poly(undecylenic acid-co-4-vinyl pyridine-co-sodium acrylate) magnetic nanoparticles with a palladium catalyst achieved a maximum yield of 96% under optimum Heck reaction conditions comprising 1 mmol iodobenzene, 1.5 mmol acrylic acid, 3 mmol base, 2 mL water, 0.09 mole% catalyst, and a reflux of 12 h [46].

**Figure 2:**
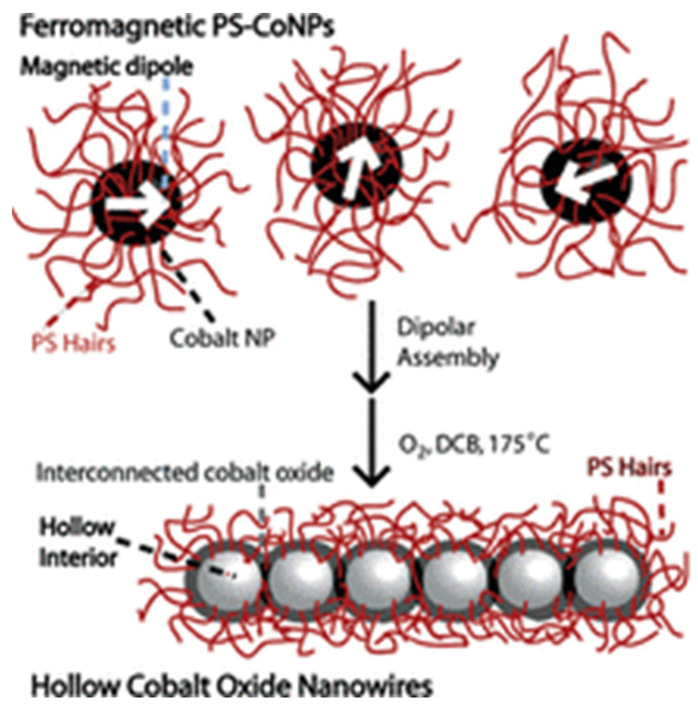
Colloidal polymerization of ferro-magnetic polystyrene-cobalt nanoparticles into cobalt oxide nanowires (reprinted with permission from [47]).

Leng et al. [2] developed an amphiphilic composite with a magnetic Fe_3_O_4_ core, a modified polyoxometalate-paired poly (ionic liquid) shell, and the surfactant dodecylamine. It was shown that the use of a heterogeneous catalyst greatly increased the efficiency and selectivity of the epoxidation of bioderived olefins with H_2_O_2_. Additionally, the catalyst showed advantages for simple magnetic recovery, recycling, and effective regeneration. It was discovered that the catalyst’s unique amphiphilic structure and the intramolecular charge transfer between the amino groups and hetero-polyanions are what account for its extraordinary performance in the epoxidation processes. Colloidal polymerization of polymer-coated ferromagnetic cobalt nanoparticles (PS-CoNPs) was performed by Keng et al. [47] in order to prepare polystyrene-coated cobalt oxide nanowires. As demonstrated in Figure 2, the dipolar assembly of PS-CoNPs and the nanoscale Kirkendall effect combined in the oxidation reaction to form interconnected cobalt oxide nanoparticles with hollow inclusions. The authors also identified a simple and dependable method for producing gram-scale quantities of well-defined polymer-coated cobalt oxide nanowires by combining a dipolar assembly and oxidation of the dipolar nanoparticles. It was also demonstrated that these nanostructured cobalt oxide materials have the potential to be used as energy storage material since they are electrically and electrochemically active.

##### Molecularly Imprinted Polymer-Based Catalyst

A new class of material, namely, molecularly imprinted polymers (MIP), with specialized properties, including high stability in harsh environments and predetermined selected cavities capable of distinguishing target molecules from their analogs based on the size, shape, and functional groups, has been widely employed as adsorbents and photocatalysts [48]. Conductive polymers, namely, polypyrrole (PPy), are promising candidates for coating material of a metal oxide catalyst owing to their superior conductivity, high polarizability, and chemical and thermal stability; they also act as photosensitizers of TiO_2_ under visible light. However, the hydrophobic nature of the composites decreases the adsorption efficiency of most organic contaminants, which can be overcome by the molecular imprinting technique [49]. TiO_2_ nanoparticles overcoated with PPy show strong affinity towards target contaminants, along with high photocatalytic activity. The system has been further improved by using magnetic titania particles coated with the MIP polymers, where the magnetic separation has an additional advantage of easy clean-up without centrifugation [50].

### 2.2. Biopolymers

The non-biodegradability of petroleum-based synthetic polymers, poor biocompatibility, and depletion of fossil fuels have necessitated the alternative path of using biopolymers for surface engineering and modification of catalyst particles. Polysaccharides, namely, chitosan, cellulose, and polyphenol derivatives, such as polydopamine (PDA), tannic acid (TA), and proteins, are the naturally occurring polymers that have been used as coating material. Particularly, the biopolymers that are widely employed as surface modification agents of catalysts include PDA, TA, and protein amyloid-like aggregates that have the ability to naturally adhere to the particle surface and form a conformal coating layer [51]. PDA-coated anodic alumina membranes impregnated with silver nanoparticles and PDOP-coated polystyrene (PS) nanotubes with highly active silver nanoparticles were used for the reduction of aromatic nitro compounds [52]. Furthermore, PDOP-coated alumina in cobalt-promoted molybdenum sulfide catalysts have been effectively used for hydrodesulfurization and hydrogenation activity by reducing the interaction between the alumina support and the catalyst metals and improving the reaction rates [53]. Polydopamine (PDA)/nickel complex-coated multiwalled carbon nanotubes are used as combustion catalysts for decomposition and combustion of energetic material [13]. Biopolymers like chitosan have been effectively employed for coating metal organic framework catalyst nanoparticles [14] and metal oxide-based nanoparticles used for electrocatalysis [15].

## 3. Method of Synthesis of Polymer-Coated Catalysts

Several methods have been employed for preparing polymer–catalyst composites for different applications. Magnetic polymer-coated nanocatalysts for triazole synthesis have been prepared by initially protecting the bare Fe_3_O_4_ nanoparticles with a silica shell to yield thiol-modified silica-coated magnetic nanoparticles (SMNP), followed by co-polymerization of the SMNP with monomeric unit vinylpyridine in the presence of a cross-linking agent [54]. Cationic porphyrin-based polymers on carbon nanotube supports used as bifunctional catalysts for cycloaddition reactions are prepared by a direct reaction of 4-pyridyl porphyrin zinc, di(1H-imidazol-1-y1) methane, and 1,4-bis bromomethyl benzene as a monomer in the presence of carbon nanotubes as support [55]. Palladium-catalyzed C-C cross coupling reactions, namely, Suzuki, Heck, and Sonogashira reactions, have been extensively investigated in various supports for utilizing the advantages of the heterogenous catalysis mode. In this respect, several researchers have used polymeric support for nanoparticles [56,57]. The one-step polycondensation technique from melamine and terephthalic acid has been applied for the preparation of a polyamide support to stabilize the palladium nanoparticles, and the conjugate catalyst with a 5.4 nm pore diameter revealed good catalytic activity and reusability in the Suzuki–Miyaura coupling reactions of aryl halides, producing a 99% yield of the desired product and high turnover frequencies of 29,400 h^−1^ [57]. A number of studies have been conducted on the preparation of polymer-coated photo- and electrocatalysts, which are described in the subsequent subsections.

### 3.1. Preparation of Polymer-Coated Photocatalysts

Large quantities of highly colored and toxic dye effluents are produced from the printing and dyeing industries. The release of these effluents causes serious pollution in the environment. The photocatalyst is usually applied in suspension mode in wastewater treatment. However, this method suffers from several drawbacks, namely, agglomeration of the photocatalyst leading to a decrease in photocatalytic activity and difficulty in the catalyst recovery process. Immobilizing the catalyst particles on a support or thin film can overcome these challenges and also improve the recyclability of the polymer–photocatalyst moiety.

Several polymers, including polyamide, polysulfone, polyethersulfone, polyvinylidene fluoride, polypropylene, polyacrylonitrile, and cellulose acetate, have been used as the support for TiO_2_-based photocatalysts. Recent studies have shown that polyethersulfone (PES) is stable under UV irradiation and is not easily degraded by the OH radicals produced in the photocatalytic reaction, making it a suitable support for photocatalytic materials [58]. PES/TiO_2_ film photocatalysts were successfully prepared by immobilizing TiO_2_ onto PES films via a phase inversion method.

For the improvement of photocatalytic activity of titanium dioxide, polyvinyl alcohol-TiO_2_ composites were also explored. Thin films of PVA/TiO_2_ were prepared by adding a measured quantity of PVA solution to a TiO_2_ solution and the mixture was casted on a glassy substrate, followed by freeze drying and heat treating to produce the conjugated polymer/TiO_2_ composites. The PVA/TiO_2_ mass ratio and the heat treatment temperature are the major controlling factors for generating composites of the correct porosity [59]. Studies show that coating photocatalysts like TiO_2_ with a sacrificial layer of polymeric material can prevent sintering of the catalyst particles during the calcination processes and retain the crystallinity of the photocatalyst without decreasing the specific surface area [60]. The photocatalytic activities of TiO_2_ essentially depend greatly on the degree of crystallization determining the percentage of anatase and rutile in the catalyst and also on the dispersibility in water. It was observed that an optimum thickness of the sacrificial polystyrene layer is necessary for arresting the aggregation of the catalyst particles. Photocatalytic particles of titania were coated with polymer shells in order to prevent the aggregation of the particles during the heat treatment. The polymer-coated titania particles were crystallized from amorphous to anatase phases by calcination at 500 °C. The crystallinity of the calcined polymer-coated particles was lower than that of titania particles calcined without polymer layers, and the photocatalytic activity of the calcined polymer-coated particles on dye removal was much higher than that of the calcined titania particles.

Superhydrophobic coatings of apatite-based photocatalysts (TiHAP) fabricated using hydrophobic organic polymers leading to the formation of composite films have shown superhydrophobicity, oil repellency, photocatalytic activity, and stability against sunlight. After undergoing hepta-deca-fluoro-decyl-trimethoxysilane (FAS) treatment, the TiHAP powders were dispersed in a mixed solvent (2-butanone and methanol) containing hydrophobic poly(methyl methacrylate) (PMMA), sonicated, and spin-coated on soda-lime glass to produce polymer composites [61].

Polyacrylic acid-coated TiO_2_ nanoparticles (NP) have been explored for their ability to generate reactive oxygen species in an aquatic environment. The polymer-TiO_2_ stock suspensions have been prepared by dispersing the powdered metal oxide in distilled water, followed by bath sonication [62]. The stock suspensions were stored at 4 °C in the dark and probe sonicated, followed by mixing to produce the polymer coated TiO_2_. Polycaprolactam (PCL) has been used for the coating of TiO_2_ nanofibers, followed by electrospinning of the PCL-TiO_2_ composites on the surface of different alloys, namely, magnesium and AM50, for controlling the degradation rate of the alloys in the human body and ensuring biocompatibility [63].

Nanohybrids of conducting polymers and zinc oxide/titanium dioxide have been extensively investigated in the purification of water because of their enhanced photocatalytic activity under solar and ultraviolet radiation [64]. The advantages of using conducting polymer/photocatalyst nanohybrids is that they absorb a wide range of visible light and act as a photosensitizer due to a lower band gap of the conducting polymers compared to ZnO. Polyaniline (PANI)/TiO_2_ composites were prepared by in situ polymerization of aniline on the surface of TiO_2_ particles. The TiO_2_ nanoparticles were dispersed in a HCl solution containing an aniline monomer and subjected to ultrasonic vibration. Ammonium peroxodisulfate dissolved in a HCl solution was added dropwise to the aniline monomer-containing solution with constant stirring and the mixture was allowed to polymerize for 5 h. After vacuum filtration and ethanol washing, the reaction mixture yielded the PANI/TiO_2_ composites [32]. Similarly, the PANI-ZnO composites prepared by in situ chemical oxidation of aniline in the presence of different amounts of diethylene glycol showed a dye removal efficiency of 90% in 60 min [65].

Coating a layer of Nafion (Nf), an anionic perfluorinated polymer with sulfonate groups, onto SiO_2_ particles can bind ruthenium and iridium polypyridine-based dye onto the SiO_2_/Nf composite through strong electrostatic interactions, resulting in a stable photocatalyst used for PMMA production [66].

Construction of a heterojunction between titanium-based metal organic framework (MOF), 125-Materials of Institute Lavoisier (MIL-125(Ti)), and conjugated polymers, namely, melamine-terephthalaldehyde, enables the hybrid photocatalyst to operate in the visible range and cleave the sp^3^ C-H bonds due to the positive valence band [67]. On the other hand, cross-linked nonconjugate polymer (PNH)-coated photo-Fenton catalysts were prepared for the photocatalytic degradation of organic pollutants. In the first step of the PNH preparation, bipyridine dicarboxylic acid (BPDCA) was added to a calculated amount of thionyl chloride and heated for the removal of the solvent to produce bipyridine dicarboxylic acid chloride (BPDCAC). Tetraazacyclotetradecan treated with triethylamine in chloroform, followed by the slow addition of a BPDCAC chloroform solution yielded a yellow PNH powder, which was dissolved in a suitable solvent and kept overnight. A facile hydrotreatment method was used for the synthesis of the Fe_2_O_3_/PNH composite, whereby PNH was dispersed in deionized water, subjected to ultrasonication, followed by the addition of an FeCl_3_ solution. The resulting solution was subjected to a hydrothermal process at 140 °C, forming a dark red powder of the composite, which was washed and dried under a vacuum [19].

The hydrothermal process was further employed to synthesize metal dichalcogenide-polyaniline composites by in situ polymerization of aniline on hydrothermally sensitized chalcogenide using an ammonium persulfate oxidizer. It is to be noted that the hydrothermal method was used due to its low cost and good control over the growth rate [68].

**Figure 3:**
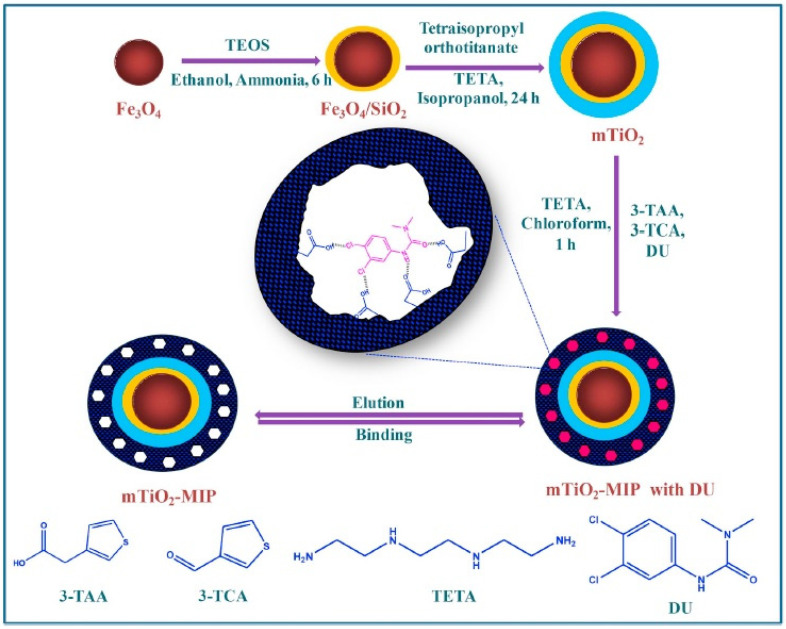
Schematic representation of the synthesis of magnetic TiO_2_-molecularly imprinted polymers (reprinted with permission from Ref. [50]).

Polyionic liquids (PIL), a subclass of polyelectrolytes, have been increasingly emerging as coating material for catalysts, as their properties can be flexibly altered by a counterion exchange. Amphiphilic composites of a dodecylamine-modified polyoxometalate (ionic liquid) shell with a magnetic Fe_3_O_4_ core have been explored for carrying out heterogenous organic transformations. In one of the studies, the ionic liquid (IL) was chemisorbed onto the Fe_3_O_4_ surface, followed by aqueous phase precipitation polymerization of the vinyl imidazole IL initiated by potassium peroxodisulfate, resulting in a PIL coating of the catalyst [2]. The schematic diagram showing the synthesis of magnetic TiO_2_-molecularly imprinted polymers is illustrated in Figure 3.

### 3.2. Preparation of Polymer-Coated Electrocatalysts

Several strategies of tailoring the electronic properties of a catalyst to actively drive the reactions of interest and improve the selectivity of the catalyst towards the desired product have been proposed. One such strategy involves the preparation of polymer/electrocatalyst nanocomposites, which are preferentially employed in electrocatalytic applications, whereby selective redox reactions are carried out at the electrified interfaces without compromising the stability and durability of the catalyst. Of the different strategies proposed, polymer coating of electrocatalysts is a useful one. Particularly, the thermochemical stability of the platinum catalyst in a methanol environment, the carbon monoxide tolerance, and the lifetime of the catalysts were three different parameters taken into consideration when selecting the polymer coating material for the electrocatalyst in a direct methanol fuel cell (DMFC). For testing the durability of the polymer-coated electrocatalysts, two different PVPA-coated electrocatalysts were synthesized using two different carbon supporting material, VulcanXC-72R (VC) and Ketjenblack (KB) [69]. Both of the carbon-supporting materials were wrapped by poly[2,2′-(2,6-pyridine)-5,5′-bibenzimidazole] (PyPBI) using sonication and the filtered and dried composite was dispersed in ethylene glycol solution. Subsequently, platinum deposition was performed by reduction of H_2_PtCl_6_. The fabricated electrocatalysts were dispersed in ethylene glycol by sonication, followed by the addition of an aqueous solution of PVPA. The solid product obtained by the ultrasonication and filtration processes was washed and dried and tested for durability and methanol tolerance.

Furthermore, a cathodic electrocatalyst was fabricated by a PVP coating on platinum nanoparticles, assisted by hydrogen bonding between PVP and polybenzimidazole (PBI). The nanoparticles were supported by carbon black (CB). The CB/PBI/Pt (composite 1) was coated with the PVP layer by dispersing it in a calculated amount of water, followed by sonication, addition of the polymer, filtration, and vacuum drying [70]. PyPBI coatings on multiwalled carbon nanotubes have further been explored as solubilizer and binder material during the loading of platinum nanoparticles. To improve the proton conductivity of the electrocatalyst, the PyPBI-wrapped MWNTs were further coated with PVPA via an acid–base reaction for high-temperature (120 °C) operation or with Nafion for low-temperature operation (70 °C). The fabricated electrocatalyst exhibited a high FC performance with a power density of 375 mW/cm^2^ (at 70 °C, 50% relative humidity using air (cathode)/H_2_(anode)), and a remarkable durability of 500,000 accelerated potential cycles with only a 5% loss of the initial FC potential and a 20% loss of the maximum power density [71]. The XPS spectra of the MWNT/PyPBI/Pt/Nafion composites and CB/PyPBI/Pt/Nafion composites showed the characteristic peaks of Nafion at 689 eV, while no Nafion peak was recognized on the Nafion-treated CB/Pt, indicating the importance of PyPBI as a binder for Nafion on the PBI-coated MWNTs and PBI-coated CB. When comparing the spectrum of the Nafion-coated MWNT/PyPBI/Pt to that of the free Nafion, a shift in the binding energy to a lower energy value by 2.5 eV was observed, which was attributed to the interaction between the sulfonic group of Nafion and nitrogen groups of PyPBI.

Polymers of intrinsic microporosity (PIM), a new group of synthetic polymers, are able to bind gases, such as hydrogen and oxygen, at the electrode surface under triphasic conditions, thereby affecting the electrocatalytic reactions. These polymers possess the inherent advantages of low molecular interaction energies in the solid state, a highly microporous structure, and high solubility in solvents and are readily coated over the surface of electrodes or catalysts by solvent evaporation. Nano-palladium electrocatalysts were prepared by electrodeposition of palladium on glassy carbon in chronoamperometry mode at a deposition potential of −0.5 V, and protected by a PIM-7 coating by drop casting. They have been employed in the reduction/oxidation of protons, reduction of oxygen, and oxidation of formic acid and methanol [72].

Polyoxometalate-based carbides are used as hydrogen evolution reaction catalysts because of their high electronegativity, which is beneficial for combining with carbon and nitrogen elements [73]. Cobalt (Co)/tungsten carbide (WC)-based electrocatalysts coated by N-doped carbon layers were effectively employed in electrocatalytic hydrogen evolution where polyethyleneimine (PEI) was used as the carbon source and polyoxometalate (POM) and cobalt ions as the metal precursors. PEI and phototungstic acid were dissolved in deionized water, followed by the addition of cobalt acetate hydrate to the solution and calcination at 800 °C for 2 h to achieve a spherical sample wrapped in a carbon shell [73].

Modifications of the surface electronic properties of nickel-based water oxidation catalysts using polymer coatings were investigated for selectively driving the reactions of interest. Using the hydrophobic PTFE-coated nickel oxide-based catalyst, the four-electron water oxidation reaction to oxygen can be altered to two electron water oxidations to H_2_O_2_, due to the collective effect of the reduced binding energy of OH* and suppressed formation of O* intermediate induced by the formation of stable Ni-CFx bonds [8].

Xia et al. [74] described an interfacial engineering strategy that produced O_2_ gas in situ to adjust the water oxidation reaction route by coating the catalyst with hydrophobic polymers. A polytetrafluoroethylene (PTFE) coating was loaded on a three-dimensional porous carbon fiber by a dip-coating process, whereby sufficient triple-phase boundaries and gas confinement for a water oxidation reaction were generated. An increase in the gas adhesive force with an increased PTFE coating provided a direct impact on the catalytic activity and selectivity during water oxidation. It was discovered that the accumulated local gaseous O_2_ could change the water oxidation reaction’s energetics in order to generate more H_2_O_2_. This change was controlled by the formation of a triple-phase boundary, at which the *OH binding energy shifts towards the volcano’s summit due to a less oxidized reaction environment and a lack of hydrogen bonding to the nearby water. The authors demonstrated a significantly higher intrinsic H_2_O to H_2_O_2_ selectivity and activity compared to that of the pristine catalyst using carbon catalysts as a model system. It was found that with an overpotential of 640 mV, the maximum H_2_O_2_ Faradaic efficiency was increased by a factor of six to 66%, allowing for the production of H_2_O_2_ at a rate of 23.4 mol per cm^2^ per minute (75.2 mA/cm^2^ partial current).

## 4. Applications of Polymer-Coated Catalysts

Polymer metal nanocomposites have been employed for reactions, namely, synthesis of triazoles, which are heterocyclic compounds useful for agrochemical and industrial applications; the transformation of carbon dioxide to cyclic carbonates; the synthesis of broad, bimodal ethylene and noble metal catalyzed oxidation; and reduction reactions. The cycloaddition reaction of terminal alkynes and azides is a powerful click reaction used for synthesizing triazole with copper immobilized in a polymer as the heterogenous catalyst. However, the separation of copper from the reaction mixture poses a limitation; to overcome the limitation, magnetic nanoparticle-based catalysts were formulated. Thiol-modified silica-coated Fe_3_O_4_ magnetic nanoparticles were copolymerized with the vinylpyridine monomer to generate the poly(4-vinylpyridine)-coated magnetic catalytic unit in the presence of a cross-linker and an initiator, followed by the incorporation of copper as the recoverable heterogenous catalyst [54]. A maximum triazole yield of 98% was obtained at optimum operating conditions of 55 °C and 3 h reaction time with 2 mol% poly(4-vinylpyridine)-coated magnetic copper catalyst in the H_2_O-*t*-BuOH solvent with a catalyst to solvent ratio 4:1. Carbon nanotube-supported cationic porphyrin-based polymers have been investigated in cycloaddition reactions of carbon dioxide with epoxides. The cationic polymer/CNT hybrid catalyst exhibited a high BET surface area of 125 m^2^/g, achieving a 99% yield of propylene carbonate and 99% selectivity of the desired product in 2.5 h during the cycloaddition reaction of CO_2_ with propylene oxide. The catalytic efficiency of the supported and unsupported composite polymer is attributed to the cooperative activation mechanism of the cationic polymer [75]. The use of copolymers for providing stabilization through steric crowding and weak binding to the particle surface through heteroatoms have been widely explored in synthesizing next-generation polymer coatings for palladium nanoparticles [6].

The catalytic activity of the polymer-coated palladium nanoparticles was evaluated in the reduction of para nitrophenol (Nip) to para-aminophenol (Amp) and the highest reaction rate of 0.003 s^−1^ was obtained at a pH of 7.4. The prepared catalysts with the highest activity in Nip reduction were tested in Suzuki cross-coupling reactions and a product yield as high as 98% was achieved. The catalyst could be recycled and reused without significant reduction in activity. Microporous network polymers prepared by the polymerization-induced phase separation (PIPS) technique were used as solid supports for palladium nanoparticles and the catalytic performance was evaluated using a Suzuki–Miyaura reaction of 4′-bromoacetophenone and phenylboronic acid in water. Capturing the nanoparticles in the microporous framework ensured suppression of loss of the catalyst and ensured greater than 90% yield of the desired product 4-acetylbiphenyl in 2 h [56].

In another study on para-nitrophenol reduction reaction using a noble metal catalyst, polymer/nanodiamond (ND) nanoparticles were used as the support for immobilizing gold, silver, platinum, and palladium nanoparticles. Nanodiamond (ND) offers chemically and mechanically robust nanoparticles with reactive functional groups at the surface, which are used to bind the thin polymer layer. The polymer/diamond-supported nanocatalysts possessed better stability compared to silica supports and remain unaltered in harsh pH conditions [76]. Polymer/nanodiamond-supported catalysts also do not suffer from the inherent disadvantage of swelling and dissolving in organic solvents, which was observed in the polymer-supported catalysts.

Incorporating surface hydrophobicity/hydrophilicity using polymers has proved to be successful in enhancing the selectivity, conversion, and recyclability of catalysts. A hydrophobic polydimethylsiloxane (PDMS) coating on metal oxides, namely, amorphous manganese oxide (AMO) and crystalline cobalt oxide (Cox), prevents water from deactivating the catalysts used in carbon monoxide oxidation [3]. Interestingly, it was observed that the polymer-coated AMO, which was coated for a span of 4 days, showed a lower CO conversion (11%) compared to the polymer-coated AMO prepared in 2 days (63% conversion), possibly because of the diffusional resistance offered by the first sample to the gases. The list of various reactions catalysed by polymer-coated catalysts and the effect of the coating on the change in catalytic activity has been summarized in Table 1. The major applications of polymer-coated catalysts have been observed in the areas of photocatalytic and electrocatalytic reactions, which are discussed in the subsequent sections.

### 4.1. Applications in Photocatalytic Reactions

Several polymers, including polyamide, polysulfone, polyethersulfone, polyvinylidene fluoride, polypropylene, polyacrylonitrile, and cellulose acetate, were used as the support for metal-based photocatalysts used for the removal of dyes and organic pollutants from effluents. The highest pollutant degradation efficiency of 98% was obtained when PES (13 wt%)-TiO_2_ (PT-13) film photocatalysts were used to degrade 10 mg/L of methyl orange solution at pH 2. The PT-13 film photocatalyst retained its high-level degradation efficiency even after 5 cycles, without being subjected to any regeneration process [78]. Improving the selectivity of TiO_2_ during pollutant degradation has been previously attempted using molecular imprinted polymers (MIP) due to their better molecular recognition ability and specific adsorption, in which a precursor is prepared via complexation of an appropriate monomer with a target pollutant, followed by coating of an MIP layer on the TiO_2_ particles via in situ polymerization The coated photocatalyst could bring about 100% decomposition in 80 min [79].

The use of conjugated polymer-doped TiO_2_ particles has shown improved photocatalytic performance under visible light and avoids the inherent tendency of agglomeration by TiO_2_ particles. While a phenol decoloration efficiency of 10–15% has been achieved using pure TiO_2_ and PVA-TiO_2_ nanocomposites, a decoloration efficiency as high as 60% is achieved in the same time using the porous conjugated PVA-coated catalyst particles. It should be mentioned that the change in morphology of the PVA-coated particles to a porous structure was attained by heat treatment at 180 °C. A coating of a sacrificial polymeric layer comprising polystyrene on TiO_2_ catalyst particles prevented sintering of the catalyst particles during calcination at 500 °C for transformation from the amorphous phase to the crystalline phase [60]. Although the crystallinity of the calcined polymer-coated catalyst was less than that of TiO_2_ calcined without polymers, about 80% dye removal was obtained using the polymer-coated catalyst, whereas only 60% removal was achieved with TiO_2_ alone.

Integrated photocatalyst-adsorbent systems were explored by designing a TiHAP system, in which calcium hydroxyapatite was partially substituted by Ti ions. Superhydrophobic composite films of PMMA and TiHAP showed excellent stability in sunlight. However, the hybrid catalysts achieved 35% decomposition of isopropyl alcohol, which was lower than that of TiO_2_ [61]. Apart from the advantages of the superhydrophobic photocatalysts mentioned earlier, the use of superhydrophobic photocatalysts has been extensively investigated due to their beneficial properties, including bacterial adhesion, superior cleaning action of the lotus effect, and water proofing. The photocatalytic activities of the superhydrophobic films deposited using a cold-walled aerosol-assisted chemical vapor deposition (AACVD) process utilizing chloroform solutions of polydimethylsiloxane and TiO_2_ nanoparticles were comparable to those of standard photocatalysts with nearly 100% decomposition of dye in 80 min [80].

Improvement of the photocatalytic activity of zinc oxide semiconductors using noble metals such as silver has been widely explored. The nanostructure containing ZnO and Ag particles exhibits novel optical, electrical, magnetic, and chemical properties. Furthermore, the Ag/ZnO nanoparticles were coated with a tri-block copolymer for high stability, enhanced photocatalytic performance, and reusability during the decomposition of organic pollutants [64].

For shifting the absorption wavelength of zinc oxide nanoparticles from the UV range to visible radiation, nanohybrids of ZnO and conducting polymers, namely, polyaniline, polyphenylendiamine, and polypyrrole, with good charge separation efficiency have been used in the degradation of organic contaminants, dyes, and toxic metals. The conducting polymers possessing a low bandgap and ZnO with a wide bandgap allow a favorable transfer of electrons between one another and high photocatalytic activity [64].

Conducting polymer/photocatalyst composites can be prepared by in situ polymerization, sol gel synthesis, and template synthesis. Polyaniline (PANI)/TiO_2_ composites, prepared by in situ polymerization of aniline on the surface of TiO_2_ particles, exhibited a degradation efficiency as high as 50% under visible light and was double that which could be achieved using TiO_2_ alone [32]. PANI-ZnO composites have shown dye removal efficiencies of 90–99% in a span of 1–5 h [65,81].

Metal dichalcogenides, namely MoSe_2_, have been used as promising photocatalytic material due to their high chemical stability, large surface area, and visible-near infrared region bandgap. However, the limitations of MoSe_2_, including a slow charge transfer, agglomeration, and high recombination rate of electrons and holes, can be overcome by the formation of MoSe_2_-PANI composites in situ polymerization of conducting polymer, such as PANI. The efficiency of dye degradation was found to be 65% for methylene blue and 94% for methyl orange, respectively, using MoSe_2_-PANI nanocomposites [68].

Increasing interest in the performance of photocatalysis in the visible range has led to the discovery of ruthenium- and iridium-based polypyridine complexes as photoredox catalysts because of their strong absorbance in the visible region, chemical stability, and favorable redox potentials [66]. Zhang et al. [66] developed a recyclable ruthenium catalyst, SiO_2_/Nf/RuL, immobilized on Nafion-coated silica, in order to photo-initiate free radical polymerization at room temperature. Under optimal conditions, photocatalytic free radical polymerization was performed using a variety of methacrylates employing -bromoisobutyrate as an initiator and a domestic fluorescent lamp as a light source, and the yield of the produced polymer achieved was as high as 3360 mg/mg catalyst. In a number of radical-mediated organic processes, this technique appears to be a quick, simple, and affordable alternative to the conventional thermal or photo-based free radical initiation methods.

Palladium (Pd) nanoparticles (NP) have been preferentially used as catalysts with high activity in the semi-hydrogenation of alkynes at low temperature, low hydrogen pressure, and excellent selectivity. Acrylate-based polymers with cleavable α-hydroxyalkylketone (HAK) substituents are used as a coating material for these catalysts, firstly, as an electron source for the photoreduction of palladium ions and secondly, as a stabilizer of the palladium ions. Masing et al. [82] presented a low-cost photoactive copolymer that may be employed as a stabilizer and reductant for the manufacture of 1.3 nm-diameter palladium nanoparticles (PdNPs). In contrast to previous procedures for preparing NP, neither the inclusion of an external reducing reagent nor an NP-stabilizing additive is necessary. The PdNP–polymer hybrid materials were found to be effective hydrogenation catalysts with excellent activity and Z-selectivity in the semi-hydrogenation of alkynes with an alkene yield of 99%. It is simple to recycle and reuse these PdNP–catalyst hybrid materials up to five times. The polymer-coated PdNPs are soluble in a variety of organic solvents with diverse polarities and were stable over a period of weeks. They were also easily isolated by simple solvent evaporation without aggregation or decomposition.

Polymer coatings are often applied to photosensitizers, such as carbon quantum dots, for enhancing hydrogen production quantum yield [83]. Hydrogen was produced photocatalytically using composites of PVP-coated carbon quantum dot (CQD) photosensitizers and a nickel nanoparticle photocatalyst when irradiated with 470 nm of light. Addition of the polymer to the CQD caused an increase in fluorescence quantum yield, thereby causing an increased amount of hydrogen production for the coated particles.

For the elimination of organic contaminants, the photo-Fenton technique, which combines advanced oxidation process (AOP) chemicals with the aid of light, has been widely used. A higher oxidation rate was attained compared to the conventional Fenton reaction, while sludge formation and total iron consumption were both significantly decreased [19]. Hematite (α-Fe_2_O_3_), a photo-Fenton material, has stronger ultraviolet (UV) to yellow area absorption than TiO_2_, and transmissions in the orange region give it its distinctive dark-red color. This allows for a higher use of sunlight than TiO_2_. Due to its great coordination affinity with Fe, a polynitrogen-containing nonconjugated cross-linked polymer called poly(1,4,8,11-cyclotetradecane [2,2-bipyridine]-5,5-dicarboxamine (PNH) has been used to synthesize a polymeric metal complex called PNH-Fe(III). By simple hydrotreatment, the polymeric metal complex is further transformed into PNH-coated metal iron nanoparticles. The N-rich polymer coating was reported to improve charge transfer between PNH and Fe_2_O_3_ and exhibits outstanding photo-Fenton activity of 90% for breaking down organic contaminants, including drugs and dye under visible light. Wider light absorption, stronger photocatalytic activity, lower catalyst loading, and improved stability are all made possible by the polymer coatings’ synergistic effects. Moreover, the catalyst can be recycled up to five times with essentially little activity loss. Fe_2_O_3_-PNH was created and characterized, and it was discovered that, in addition to being an excellent photocatalyst, it also made it possible to use nonconjugated polymers in the photocatalysis process. Copper-doped mesoporous polyimide nanocomposites, developed via in situ doping and hydrothermal polymerization, were employed for refractory pollutant degradation in a pH range of 2.6–10.4. The catalyst exhibited a Fenton-like activity of 70–80% degradation of most of the pollutants and a reaction rate 15–20 times higher than conventional uncoated Fenton catalysts [84].

The reactions catalysed by polymer-coated photocatalysts and the effect of the coating on the catalytic activity have been summarized in Table 2.

### 4.2. Applications of Polymer-Coated Electrocatalyst

Polymer-coated electrocatalysts have diverse applications, namely, oxygen reduction reaction (ORR), hydrogen evolution reaction (HER), and redox transformations of organic molecules.

**Figure 4:**
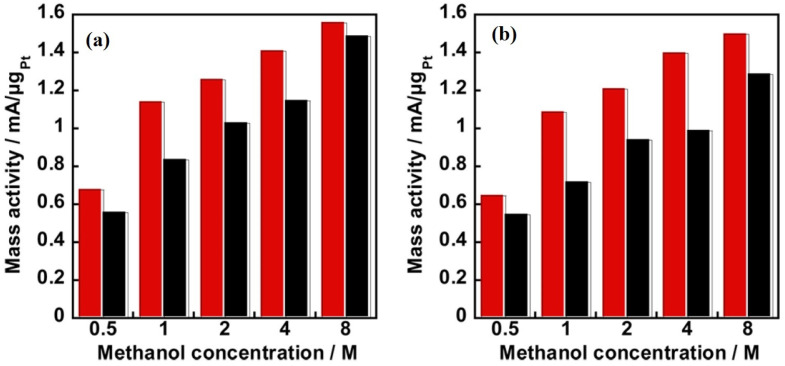
Mass activities of the catalyst Ketjenblack (KB)/ Poly[2,2’-(2,6-pyridine)-5,5′-bibenzimidazole](PyPBI)/Platinum(Pt)/poly(vinylphosphonic acid)(PVPA) (red column) and Vulcan(VC)/Poly[2,2′-(2,6-pyridine)-5,5’-bibenzimidazole](PyPBI)/Platinum(Pt)/poly(vinylphosphonic acid)(PVPA) (black column) as a function of methanol concentration (**a**) before and (**b**) after the durability test (reprinted with permission from Ref. [69]).

As discussed earlier, durability, methanol, and carbon monoxide tolerance are key factors that have been considered while designing an electrocatalyst for DMFC. A comparative study conducted on PVPA-coated electrocatalysts on two different carbon supports [69] shows that the composite catalyst KB/PyPBI/Pt/PVPA (composite 1) with a higher electrochemical surface area (ECSA) shows a higher durability (7% loss in ECSA) under the potential cycling from 1.0 to 1.5 V vs. the reference hydrogen electrode (RHE) compared to that of the VC/PyPBI/Pt/PVPA (composite 2), which showed a 20% loss in ECSA after the durability tests. Meanwhile, composite 1 showed a higher CO tolerance before and after the durability test compared to that of composite 2, especially under a very high methanol concentration (4 M and 8 M), which is close to the practical application of the DMFC. The metric used to determine the CO tolerance of the catalysts was the ratio I_f_/I_b_, where I_f_ and I_b_ are the anodic and reverse anodic peaks, respectively. The observed higher CO tolerance was due to the higher amount of the coating polymer PVPA (14.6 wt %) in composite 1 caused by the higher specific surface area of the KB (1232 m^2^/g) compared to the VC (235 m^2^/g). The mass activities of the electrocatalysts in the methanol oxidation reaction before and after the durability tests (Figure 4) indicate the superior durability of composite 1.

A platinum catalyst sandwiched between two polymer layers, namely, PVP and PyPBI, showed a 14% reduction in ECSA compared to the non-coated catalyst (38% loss in ECSA) after durability tests, indicating a higher stability of platinum in the coated catalyst. The composite catalyst was supported by carbon black. The composite catalyst further achieved higher oxygen reduction reaction (ORR) activity under different methanol concentrations in the electrolyte. The higher methanol tolerance was possibly due to lower accessibility of methanol to the platinum catalyst through the PVP layer, but a negligible effect on oxygen diffusion. The percentage of the intermediate product in the oxygen reduction reaction, namely, H_2_O_2_, needs to be minimized for the complete reduction of oxygen to H_2_O. With a high methanol concentration of 2 M, the composite catalyst showed only 2% H_2_O_2_ generation compared to commercial catalysts, which generated 21% H_2_O_2_. PVP coating produced enhanced platinum stability, improved methanol tolerance, and exhibited higher activity of ORR reactions [70].

**Figure 5:**
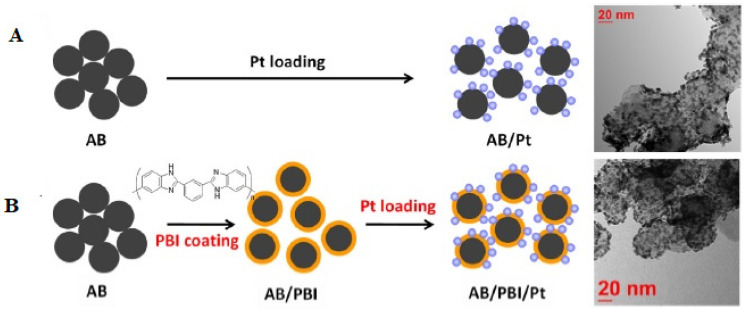
Schematic of the preparation of (**A**). Platinum loaded on acetylene black (AB/Pt) and (**B**) Platinum loaded on polybenzimidazole-coated acetylene black (AB/PBI/Pt). TEM images of AB/Pt and AB/PBI/Pt are also displayed (reprinted with permission from [86]).

A different carbon support material, namely, acetylene black (AB), wrapped with polybenzimidazole (PyPBI) with deposited platinum nanoparticles [86] indicated an 8% reduction in the maximum power density of the membrane-electrode assembly using polybenzimidazole-coated acetylene black and a 34% reduction using non-coated acetylene black. A comparison of the Pt morphology before and after the accelerated durability tests showed that the polybenzimidazole coating improved the Pt dispersion stability. During the accelerated durability tests, Pt agglomeration was also visible in the TEM image for acetylene black/Pt, showing that sintering, as well as Ostwald ripening happened for acetylene black/Pt. The preparation of the catalysts with the SEM images are shown in Figure 5.

The development of double polymer-coated carbon nanotube-based electrocatalysts has also been investigated to achieve durability in the harsh environment. For improving the proton conductivity of the electrocatalyst, PyPBI-coated multiwalled carbon nanotubes loaded with platinum nanoparticles were further wrapped with a proton conducting ionomer, PVPA, for high temperature operations, and with Nafion for low temperature operations. The Nafion-coated electrocatalysts achieved a high-power density of 350 mV/cm^2^ and underwent negligible reduction (20%) in the power density after 500,000 potential cycles, indicating high durability. The XPS spectra of the MWNT/PyPBI/Pt/Nafion composites and the CB/PyPBI/Pt/Nafion composites showed the characteristic peaks of Nafion at 689 eV, while no Nafion peak was recognized in the Nafion-treated commercial CB/Pt. These results demonstrate the importance of PyPBI (a basic polymer) as a binder to attach the Nafion (an acidic polymer) on the PBI-coated MWNTs and PBI-coated CB. When comparing the spectrum of the Nafion-coated MWNT/PyPBI/Pt to that of the free Nafion, a shift in the binding energy to a lower energy value by 2.5 eV was observed, which was attributed to the interaction between the sulfonic group of Nafion and the nitrogen groups of PyPBI. A similar observation was reported for the polyaniline-modified poly(styrene sulfonate) membranes. In addition, a new peak appeared around 401.6 eV, which was attributed to the NH+ group on the MWNT/PyPBI/Pt/Nafion, supporting the interaction of the PyPBI with the SO_3_ moieties of Nafion [71].

Coating PIM on electrocatalysts generates stable microporous films that facilitate diffusion of smaller molecules, such as hydroquinone and methanol, through the films. The oxidation of formic acid at the electrodeposited non-coated palladium films and PIM-coated palladium films demonstrates an enhanced effect of PIM-7 on hydrogen production from formic acid, which was attributed to the absorption of hydrogen into the PIM-7 film without catalyst blocking at the palladium surface [72]. The enhancement in oxidation in the PIM-coated catalyst has been observed in a two-fold increase in oxidation currents in the PIM-coated catalyst compared to the uncoated catalyst. However, the PIM coating hinders the methanol oxidation, as is evident from the decrease in the current density in the presence of a PIM-coated catalyst.

Cobalt (Co)/tungsten carbide (WC)-based electrocatalysts coated by N-doped carbon layers were effectively employed in the electrocatalytic hydrogen evolution where polyethyleneimine (PEI) was used as the carbon source and polyoxometalate (POM) and cobalt ions as the metal precursors. In acidic and alkaline media, the N-doped carbon-coated catalyst showed highly favorable hydrogen evolution activity with overpotentials of 158 mV and 178 mV at 10 mA/cm^2^ because of the cooperation between Co and WC, the porous structure, and the carbon layers [87].

Modifications of the surface electronic properties of nickel-based water oxidation catalysts using polymer coatings were investigated for selectively driving reactions of interest. Using the hydrophobic PTFE-coated nickel oxide-based catalyst, the four-electron water oxidation reaction to oxygen can be altered to two electron water oxidations to H_2_O_2_ due to the collective effect of the reduced binding energy of OH* and the suppressed formation of the O* intermediate induced by the formation of stable Ni-CF_x_ bonds [8]. Xia et al. [74] described an interfacial engineering strategy that produced O_2_ gas in situ to adjust the water oxidation reaction route by coating the catalyst with hydrophobic polymers. It was discovered that accumulated local gaseous O_2_ could change the water oxidation reaction’s energetics in order to generate more H_2_O_2_. This change was controlled by the formation of a triple-phase boundary, at which the *OH binding energy shifts towards the volcano’s summit due to a less oxidized reaction environment and a lack of hydrogen bonding to the nearby water. The authors demonstrated a significantly higher intrinsic H_2_O to H_2_O_2_ selectivity and activity compared to that of the pristine catalyst using carbon catalysts as a model system. It was found that with an overpotential of 640 mV, the maximum H_2_O_2_ Faradaic efficiency was increased by a factor of six to 66%, allowing for the production of H_2_O_2_ at a rate of 23.4 mol per cm^2^ per minute (75.2 mA/cm^2^ partial current).

### 4.3. Coating of Catalysts for Photoelectrochemical Reactions

Polymer-coated catalysts have been employed widely in photoelectrochemical reactions. Coordination polymers, namely, covalent organic frameworks and metal organic frameworks, have emerged as a class of material suitable for functional coatings of photoelectrochemical catalysts [1]. Toe et al. [88] revealed a technique for improving a ZnO semiconductor’s photoelectrochemical performance by simultaneously loading Pt deposits and a polymer coating onto the particles. By platinum cation photoreduction and phenol photooxidative polymerization, zinc oxide was deposited together with platinum metal and a layer of oxidized polymeric species. The researchers indirectly demonstrated the viability of photocatalytic polymerization using ZnO by leveraging the redox ability of ZnO to reduce platinum cations (PtCl6)^2−^ into metallic Pt while promoting the development of a thin polymer layer upon irradiation. The addition of Pt as an electron sink to promote charge separation served as an example of a constructive improvement for photoelectrochemical performance. The maximum photocurrent density obtained using the poly/Pt/ZnO composite was 14 µA/cm^2^ compared to 10 µA/cm^2^ for the Pt/ZnO composite without a polymer coating and 8 µA/cm^2^ for bare ZnO. Thus, the photoelectrochemical performances of the photocatalysts are in the following order: poly/Pt/ZnO > Pt/ZnO > bare ZnO, demonstrating the need for platinum and polymer coatings in improving the charge separation within ZnO.

A BiVO_4_ photoanode coated with poly(p-phenylene pyromellitimide) (PI) polymeric film shows a 2.5 times enhancement in photoelectrochemical activity compared to the pristine photoanode, as well as excellent stability in neutral and alkaline electrolytes. The improvement in the photoelectrochemical activity is attributed to the improvement of the surface reaching hole reaction efficiency and the surface carrier separation efficiency. Here, the PI acts both as the catalytic layer for promoting the water oxidation reaction and, simultaneously, as a protective layer in preventing the semiconductor from being corroded. The PI layer was coated on the photoanode by in situ thermal polymerization with pyromellitic dianhydride and p-phenylenediamine as precursors. An optimum PI coating of 20 nm showed a maximum photocurrent density of 3 mA/cm^2^ at 1.23 V [89].

Polythiophene, a p-type semiconductor, is unable to carry out n-type processes such as CO_2_ reduction on its own. However, when illuminated, it can transfer electrons to the catalyst from the least occupied molecular orbital (LUMO) level, while the hole in the highest occupied molecular orbital (HOMO) recombines with an electron coming from the electrode to complete the circuit. Rhenium-based complexes with bipyridine (bi-Py) ligands show excellent properties in terms of activity and a longer lifetime in these reactions. These properties have been explored for enhancing the catalytic activities of rhenium complexes. A rhenium-containing monomer [3HRe(bpy)(CO)_3_Cl-Th] was polymerized in a one-compartment cell containing boron trifluoride diethyl etherate (BFEE) as the supporting electrolyte in a three-electrode system. The surface of the platinum electrode was fully covered with a thin layer of the polymer film and 33% of Faradaic efficiency was achieved at −1150 mV versus NHE [90].

Electrocatalytic and photoelectrochemical applications employ stable surface immobilization on high band gap oxide semiconductors. Electropolymerization is a technique used for on-surface preparation of assemblies by electrochemically induced C–C coupling [91]. The idea has been implemented in preparing films of vinyl functionalized complexes (poly1) on glassy carbon (GC) and metal oxide electrodes and in a bilayer catalyst-chromophore assembly on metal oxides, used in dye-sensitized photoelctrosynthetic cell for water splitting. The Faradaic efficiency for oxygen production by water oxidation was 62% at pH 7, while the turnover frequency of oxygen was an 8.5 mol O_2_/mol catalyst for GC complexes, indicating significant catalytic activity of the polymeric film [92]. The study above was extended further to prepare a chromophore-catalyst RuPdvb^2+^-poly1 on nanocrystalline TiO_2_, which showed stable, significantly high photocurrents (60 µA/cm^2^–50 µA/cm^2^) over a span of 210 s, indicating sustained light-driven water oxidation. Here, in the chromophore RuPdvb^2+^, Ru indicates ruthenium and Pdvb-phosphonic acid-based divinyl bipyridine group. Thin film overlayers of PMMA have been used to stabilize ruthenium (Ru) (II) polypyridyl (Py) complexes bound to mesoporous nanoparticle metal (TiO_2_) films over a pH range 1–12 by dip coating. Photostability measurements indicated less than a 30% loss of the Ru–Py complex from the metal oxide during the 16 h irradiation period and an exponential decrease in the desorption rate constant with an increase in the PMMA layer thickness [93]. Cobalt dithiolene polymer coated on a glassy carbon surface or silicon provides a metal organic surface (MOS) and has been tested for solar-driven hydrogen production from water. The MOS could produce photocurrents up to 3.8 mA/cm^2^ at 0 V versus the reference hydrogen electrode [25].

### 4.4. Biopolymer Coating on a Catalyst

The disadvantages of non-biodegradability and undesirable biocompatibility have paved the path for the development of biopolymeric coatings for catalysts. Natural polysaccharide polymers (chitosan and cellulose), polyphenol-based biopolymers (polydopamine and tannic acid), and proteins have been used in the surface modification of particles [51]. A one-pot synthesis method of 10–20 nm sized chitosan (CS)-coated copper oxide nano electrocatalysts were prepared for CO_2_ reduction in aqueous media and the coated catalyst successfully retarded the hydrogen evolution reaction [15].

**Figure 6:**
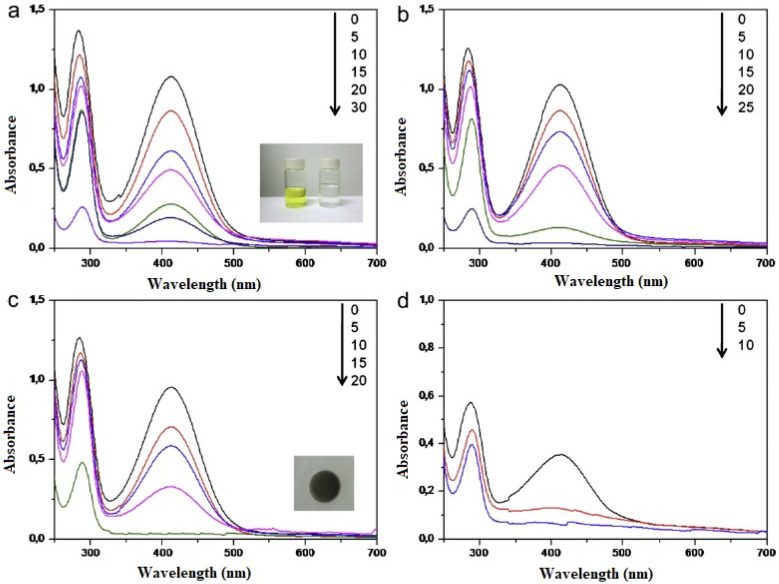
UV-Vis spectra of aqueous solutions of ortho-nitroaniline and sodium borohydride (NaBH_4_) in the presence of (**a**) silver nanoparticle-polydopamine-AAO (20 nm) with photos of o-nitroaniline before and after reduction in the inset (**b**) silver nanoparticle-polydopamine-AAO (100 nm) (**c**) silver nanoparticle-polydopamine-AAO(200 nm) inset: photo of silver nanoparticle-polydopamine-AAO (**d**) silver nanoparticle-polydopamine-polystyrene nanotube (reprinted with permission from [52]). The coloured lines in each figure indicate the absorbance (concentration data) at (**a**) 0, 5, 10, 15, 20, 30 min (**b**) 0, 5, 10, 15, 20, 25 min (**c**) 0, 5,1 0, 15, 20 min and (**d**) 0, 5, 10 min.

Polydopamine coatings prepared by oxidative polymerization of dopamine in alkaline aqueous media have been used as surface modification agents for a wide variety of solid surfaces, namely, metal oxides, ceramics, and synthetic polymers. For instance, anodic alumina membranes (AAO) of pore size between 20–200 nm were immersed in dopamine solutions for various intervals, following which the polydopamine-coated membranes were transferred to a silver nitrate solution for growing the silver nanoparticles. Following standard protocols, polydopamine-coated polystyrene nanotubes with immobilized silver nanoparticles were also prepared. The results of the NaBH_4_-assisted reduction of o-nitroaniline using the supported membrane and the nanotubes indicated a faster reaction (completion in 10 min) in the case of the nanotubes, owing to an increase in the surface area and an increase in the density of the immobilized silver nanoparticles on the inner and outer walls of the nanotubes [52] (Figure 6).

Metal organic frameworks (MOF) coated with chitosan formed core–shell structure nanoparticles with acidic and basic sites and were used for catalyzing tandem reactions, such as a deacetalization–Knoevenagel condensation reaction. An in situ growth method was used for coating the surface of MIL-101 (Cr) with chitosan and preparing the MIL-101–chitosan nanoparticles. It was observed that the yield of the final product of the condensation reaction, namely, 2-benzylidenemalononitrile, was enhanced with the increase in chitosan loading, with the maximum yield of 99% being obtained at MIL-101 (Cr):CS at 2.86:1 due to the most favourable synergistic catalysis between the acidic and basic sites [14].

Nickel–molybdenum (Ni-Mo) catalysts on an Al_2_O_3_ support exhibit limited desulfurization performances due to their strong metal/support interaction resulting in the formation of species that are difficult to be sulfided. However, coating of the alumina support with the sacrificial carbon layer obtained from the carbonization of polydopamine could promote the reduction and degree of sulfidation of the loaded phase. The HDS catalysts were prepared by loading Ni and Mo on the alumina-PDA supports via the incipient wetness impregnation method, followed by drying at 120 °C and calcinations in flowing air at 500 °C for 2 h. It was observed that at 300 °C, 2 MPa hydrogen pressure, and liquid hourly space velocity of 12 h^−1^, the percentage of HDS conversion and the yield of biphenyl increased with increase in the polydopamine concentration and achieved a maximum at an optimum value of the dopamine coating [94]. Studies on cobalt (Co)–molybdenum sulfide (MoS) catalysts on polydopamine-coated alumina supports indicate an increase in the catalytic activity in the hydrogenation of toluene and the hydrodesulfurization of gas oil feed attributed to the increase of accessibility of the active sites due to reduced MoS_2_ aggregation and a decrease in metal–support interactions due to the carbonaceous intermediate layer [53].

## 5. Influence of Polymers on Catalytic Behavior

### 5.1. Effect on Activity, Selectivity, and Turnover Number/Frequency

Polymeric materials have been used as support matrices for the immobilization of catalysts, for preparing metal–polymer-based cooperative catalysts with multiple active sites, for ensuring stability in photocatalytic reactions, and improving the selectivity of the photocatalyst during pollutant removal. In electrocatalytic and electrophotosynthetic reactions, polymeric material is essential for ensuring the durability of the catalyst after several cycles in the fuel cell operation. Furthermore, the coating material also plays an important role in ensuring the recyclability and reusability of the catalyst. For instance, in heterogenous copper catalyzed azide-alkyne cycloaddition reactions, namely, triazole synthesis reactions, covalently linked polymers have been used as substrates. Polystyrene-based material with dimethylaminomethyl moieties acting as a ligand and a base at the same time have been used to promote the catalytic reactions [54]. Cross-linked polymeric ionic liquids have been designed to improve the catalytic efficiency and reduce the leaching of copper from the catalyst moiety. Copper catalysts in polymeric substrates have obtained a 99% yield in cycloaddition reactions and have been reused 10 times without a loss in activity [95]. Copper catalysts immobilized on poly(N-isopropylacrylamide-co-4-vinylpyridine) hydrogels yield 94% triazoles with a turnover frequency of 9.4 with negligible loss in activity [77].

Bifunctional polymers co-incorporated with porphyrin-zinc as Lewis acid sites and bromide as nucleophiles for the cycloaddition of CO_2_ to epoxides indicate synergistic effects between the Lewis acid sites and nucleophiles with 99% conversion, 99% selectivity, and a turnover frequency (TOF) of 12,000 h^−1^ for the cycloaddition of CO_2_ and propylene oxide [55]. The cation porphyrin bifunctional catalyst on a carbon nanotube support produced a 99% yield and selectivity with a maximum TOF of 2602 h^−1^. Porous organic catalysts prepared via meta porphyrin compounds and phosphonium salt monomers resulted in 99% conversion and 99% selectivity during the cycloaddition of CO_2_ with epichlorhydrin. However, the percentage of metallic species governed the percentage of conversion. For the percentage of metallic species, only 24% conversion was achieved. The combination of Lewis acid and Lewis basic sites at the molecular level resulted in the high percentage conversion and selectivity [96]. No loss in activity was observed after 5 cycles of operation. However, since the performance of the catalysts was sensitive to the temperature and the metallic content, a thorough investigation is necessary regarding the performance of the catalysts under different environmental conditions.

The photocatalytic activity of polymer–metal oxide thin film catalysts (PES/TiO_2_) increased with an increase in the amount of immobilized TiO_2_, achieving a maximum degradation efficiency of 80% at 13 wt%, which decreased at a higher TiO_2_ content due to the agglomerated TiO_2_ particles. The efficiency of the film photocatalyst is attributed to the porous structure, which is necessary for light reflection and for allowing passage to the dye molecule to adsorb on TiO_2_. The film catalyst with 13% TiO_2_ was stable against UV radiation and in acidic and basic solutions, and retained its activity after 5 cycles, indicating that the polymer layer [78] provided a benefit. Molecular imprinted polymer-coated catalysts with water-compatible molecularly imprinted polymers (CMIP) are an emerging class of polymers that have been employed in photocatalytic reactions. However, the choice of a suitable functional monomer and binding capacity between the monomer and template are critical when preparing these catalysts [85]. Novel thiol functionalized CMIP-coated TiO_2_ was prepared using 2,4-dinitrophenol as a template and o-phenylenediamine (OPDA) as a functional monomer and cross-linker. The removal efficiency of 2,4-dinitrophenol using the CMIP-coated catalyst was 75%, which decreased to 65% after 5 cycles. However, further investigations are needed in this area to study the degradation efficiencies and recyclability in different pollutants and the effect of coating thickness on the catalytic activity. In other instances of photocatalytic reactions, a polymer coating was effective in the degradation of pollutants with high degradation efficiency of 70–90% without considerable loss in activity after several reuses [64,80]. Furthermore, conducting polymer–metal oxide composites have the additional advantage of good charge separation efficiency, operability under visible light, and a degradation efficiency as high as 50% in visible light [65]. Polymer-coated electrocatalysts have shown considerable durability and carbon monoxide and methanol tolerance. The activity of the electrocatalysts have been on par with the non-coated catalysts [69,70]. The studies above have been optimized with respect to polymer coating thickness, percentage of catalyst, pH of the reaction medium, reaction temperature, and reaction time. However, although the studies have reported negligible diffusional resistances to the diffusion of the reactants and products into and out of the polymer matrices, a detailed study on diffusional resistances to the transport of reactants and products through the polymer layer is warranted. The following section discusses a few studies that have been carried out on mass transfer limitations in reactions catalyzed by polymer–catalyst composites.

### 5.2. Mass Transfer Limitations

The interplay between mass transport limitations and the rate of reaction in polymer-coated catalysts are important factors governing the percentage conversion and yield of the product. Specifically, the mass transfer limitations have been studied in detail in nanoreactors comprising catalysts coated with thermo-responsive polymers. The effects of mass transport limitations on reaction kinetics were investigated in reactions catalyzed by uncoated palladium/alumina catalysts and temperature responsive nanoreactors formed by catalysts coated with N-isopropylacrylamide polymer (p-NIPAM) brushes [97,98]. A key reaction studied in this respect included the nitrite hydrogenation reaction, a step in the removal of nitrates and nitrites from drinking water. The findings of the first study revealed that low selectivity towards ammonia using the p-NIPAM-coated catalytic reaction could not be attributed to external or internal mass transfer limitations, as mass transfer limitations on the bulky nitrite ions would favourably lead to high ammonia formation rates with an increase in the H/N ratio. Moreover, in mass transfer-limited processes, the observed reaction orders for hydrogen and nitrites should be one since molecular diffusion is a first order process. However, in the present case, the reaction order was zero. Thus, the effect of mass transfer limitations in the hydrogenation reaction catalyzed with polymer-coated catalysts were ruled out and modification of the solvation environment induced by p-NIPAM around the active site was attributed to be the reason behind the poor ammonia selectivity.

The one-dimensional governing differential equation for mass transfer inside a porous catalyst was based on the Nernst Planck diffusion model, which was set up to understand the effect of catalyst particle size and polymeric brushes on the transport of reactants and products into and out of the particle. The model was used to explain the mass transfer effects on both coated and uncoated catalysts. The particle size was increased from 0–38 μm to 38–100 μm, and the change in particle size was incorporated into the reaction kinetics. In the uncoated catalyst particles, the effect of the increase in particle size from a 0–38 μm range to a 38–100 μm range had a pronounced effect on the concentration drop of the reactants, namely, NO_2-_ and H_2_ inside the catalyst particle. In p-NIPAM-coated catalyst, the concentration profiles were found to be similar to that of the uncoated catalyst when the particle size was in the range 0–38 μm, whereas for larger particles, significant drops in the reaction rates were observed in the coated catalysts. The coating decreases the activity of the larger catalyst particles, resulting in decreased apparent activation energy at temperatures above the low crystal solubility point (LCST) with a reduction in the turnover number based on NO_2_^−^ from 4.5 units to 1.5 units above LCST. This can be attributed to the collapse of the thermoresponsive polymer at the temperature above LCST, due to which, on one hand, the transport is enhanced due to opening of pores; on the other hand, the molecular diffusion is hindered by the blockage of pore constrictions. The pore constrictions were responsible for the inflection point observed in the Arrhenius plot at the LCST.

Nanoreactors fabricated with temperature-responsive p-NIPAM-coated with mesoporous silica hollow spheres on the external pore mouth and with gold (Au) nanoparticles on the internal pore mouth showed excellent catalytic activity with a turnover frequency of 14.8 h^−1^ at temperatures above LCST [98]. However, above LCST, the TOF dropped to 2.4 h^−1^. The sharp difference in the catalytic activities of p-NIPAM/Au mesoporous silica below and above LCST was attributed to the thermoresponsive behavior of the polymer. At low temperatures, the p-NIPAM chains adopt an extended conformation, allowing easy diffusion of the reactants and products to the pore mouth of the silica, where the reaction occurs at the surface of the Au nanoparticles. However, at a higher temperature, the dense hydrophobic polymer layer hinders the diffusion of the reactants into the nanoreactor, resulting in an inhibitory effect on the reaction kinetics. This occurs because, above the LCST, the collapsed polymer blocks most of the diffusional path of the reagent to the nanoreactor, whereby the diffusion of the reagents becomes the rate-determining step, resulting in pseudo-zero order kinetics.

## 6. Conclusions and Perspective

The applications of polymeric coatings on catalyst surfaces have emerged as promising strategies for controlling the catalyst surface properties, namely, improving the surface area per unit mass of catalyst, enhancing the catalytic activity and selectivity, increasing the number of cycles of operation, and improving the turnover number and the thermal and mechanical stability of the catalyst. The catalytic activities are measured in terms of percentage reduction in pollutant concentration in wastewater during photocatalytic treatment, percentage conversion, yield, and selectivity of the desired product in oxidation, reduction, hydrodesulfurization, and cycloaddition reactions, oxygen evolution, and oxygen reduction reactions during electrocatalysis. Various strategies can be used for coating the surfaces of catalysts used in important applications and their catalytic activities can be improved by manifolds through polymer coating. Novel polymeric material, namely, molecularly imprinted polymers with high selectivity for the target compounds, porphyrin-based polymers, single atom catalysts, and conducting polymers with their own advantages have been employed as coating materials. Many studies have reported the kinetics of the reactions under coated and non-coated conditions, and optimum reaction conditions are required to achieve maximum productivity. However, extensive investigations should be carried out to identify the optimum coating thickness, catalyst loading, reaction temperature, pressure, catalyst-to-reactant ratio, solvent concentration, and pH of the medium for carrying out the reactions in the presence of the uncoated and polymer-coated catalysts. From an engineering point of view, the optimum conditions need to be determined to obtain a high yield of the desired product/high rate of degradation of pollutants and maximum selectivity for the target product. Detailed kinetic studies of the polymer-coated catalysts and the effect of mass transfer limitations on the reaction rate and product yield need to be performed for developing robust industrially applicable heterogenous polymer-catalyst nanocomposite systems. In-depth investigations are necessary to determine the performance of polymer-coated catalysts under varying flowrates, stirring rates, and flow patterns in lab-scale and industrial scale reactors. In-depth understanding of the interfacial chemistry between the polymeric material and catalyst particles, managing the porosity and other surface properties, and understanding the need for functionalizing the polymer surface with chemicals for better performance and stability are some of the factors that will lead to the design of robust polymer/catalyst composites with long lifetimes. Computational modelling will further aid in the selection and design of the composites. Several challenges exist in the area of polymer-coated catalysts. For applications in redox catalysis with coordination polymers, a common challenge observed is facilitating charge transfer to catalytic centers positioned throughout the framework. A strategy developed for improving electron mobilities includes incorporation of conductive guest species. Controlling the film thickness and positioning the redox sites in MOFs can reduce the charge transfer issues [1]. Managing the interfacial chemistry with polymeric coatings in different applications has been achieved by chemically tailoring material for carrying out essential chemical transformations. However, finding new and efficient ways of interfacing material on electrode or catalyst surfaces and characterizing the hybrid material require innovations in the area of surface chemistry, catalysts, and polymer science in developing new polymeric materials catering to the need. However, to avoid the trial-and-error method of selecting the material, computational and theoretical modelling will be helpful in providing understandings of the structure–function relationships governing the assemblies. Similarly, in the area of dye-sensitized photoelectrochemical cells, chromophore-catalyst assemblies have been investigated and water oxidation catalysts with a polymer overlayer have been fabricated for maximizing water oxidation rates. However, challenges that remain to be addressed are the requirement of continuous manipulation of the molecular and interfacial structure to maximize the efficiency of light-driven water splitting, selecting material suitable for operating in the visible region wavelength, achieving maximum solar efficiencies, stabilizing the surface binding and stability of the assemblies for long-term performance, and incorporating organic dye as chromophores and first-row transition metals as catalysts [91].

## Figures and Tables

**Table 1:** Results on yield, selectivity, and turnover frequency in reactions catalyzed by polymer-coated catalysts.

Reaction Catalyzed	Catalyst Used	Turnover Frequency/Site Time Yield	Reaction Rate/Reaction Conditions	Yield%	Reference
Suzuku coupling reaction and para-nitrophenol reduction	Poly (ethylene oxide)-b-poly[2,3-(diisopropylamino)ethyl methacrylate as coating material and palladium nanoparticle catalyst		3.12 × 10^−2^ s^−1^/pH 5.9	98	[6]
1,2,3-triazole from terminal alkynes and azides	Poly(4-vinylpyridine) coated magnetic Fe_3_O_4_ nanoparticles with copper	-	-/55 °C, catalyst: solvent 4:1	98	[54]
Suzuki–Miyaura reaction producing 4-acetylbiphenyl	Poly(amidoamine) dendrimers/palladium nanoparticle	10,000	-/80 °C, 0.01 molar equivalent polymer	100	[57]
Cycloaddition reaction of propylene oxide and carbon dioxide	Zinc–porphyrin polymers coated on carbon nanotubes	2602	-/120 °C, 1.5 MPa, 2.5 h	99	[75]
Para-nitrophenol reduction	Polymer/nanodiamond composite	STY 00018	/298 K	>95	[76]
Azide-alkyne cycloaddition	Copper/poly(N-isopropylacrylamide-co-4-vinylpyridine)	9.4	5 mole% catalyst, 303 K	94	[77]

**Table 2:** Photocatalytic reactions with polymer-coated catalysts.

Reaction Type	Catalyst Used	Percentage Conversion	Rate Constant (min^−1^)	Recyclability (Number of Cycles)	Reference
Removal of tetracycline	Poly(1,4,8,11-cyclotetradecane [2,2-bipyridine]-5,5-dicarboxamine)-Fe_2_O_3_	90	-		[19]
Diuron degradation	Molecularly imprinted polymer nanocomposite with embedded magnetic TiO_2_	99	0.09	5	[50]
Phenol degradation	Porous PVA-coated TiO_2_	60	-	-	[59]
Methyl orange degradation	PES/TiO_2_ film	90	0.00412	5	[78]
Methylene blue and malachite green degradation	Polyaniline/ZnO nanocomposite	99	0.024		[81]
Alkyne semihydrogenation	Acrylate polymer-coated palladium nanoparticles	99	-	4	[82]
Organic pollutant degradation	Copper-doped polyimide nanocomposites	80–100		6	[84]
Photodegradation of dinitrophenol	Molecularly imprinted thiol functionalized TiO_2_	74.5	0.0067	5	[85]

## Data Availability

Not applicable.
